# Distinct Human and Mouse Membrane Trafficking Systems for Sweet Taste Receptors T1r2 and T1r3

**DOI:** 10.1371/journal.pone.0100425

**Published:** 2014-07-16

**Authors:** Madoka Shimizu, Masao Goto, Takayuki Kawai, Atsuko Yamashita, Yuko Kusakabe

**Affiliations:** 1 National Food Research Institute, NARO, Tsukuba, Ibaraki, Japan; 2 RIKEN SPring-8 Center, Sayo, Hyogo, Japan; 3 Graduate School of Medicine, Dentistry and Pharmaceutical Sciences, Okayama University, Okayama, Japan; German Institute for Human Nutrition, Germany

## Abstract

The sweet taste receptors T1r2 and T1r3 are included in the T1r taste receptor family that belongs to class C of the G protein-coupled receptors. Heterodimerization of T1r2 and T1r3 is required for the perception of sweet substances, but little is known about the mechanisms underlying this heterodimerization, including membrane trafficking. We developed tagged mouse T1r2 and T1r3, and human T1R2 and T1R3 and evaluated membrane trafficking in human embryonic kidney 293 (HEK293) cells. We found that human T1R3 surface expression was only observed when human T1R3 was coexpressed with human T1R2, whereas mouse T1r3 was expressed without mouse T1r2 expression. A domain-swapped chimera and truncated human T1R3 mutant showed that the Venus flytrap module and cysteine-rich domain (CRD) of human T1R3 contain a region related to the inhibition of human T1R3 membrane trafficking and coordinated regulation of human T1R3 membrane trafficking. We also found that the Venus flytrap module of both human T1R2 and T1R3 are needed for membrane trafficking, suggesting that the coexpression of human T1R2 and T1R3 is required for this event. These results suggest that the Venus flytrap module and CRD receive taste substances and play roles in membrane trafficking of human T1R2 and T1R3. These features are different from those of mouse receptors, indicating that human T1R2 and T1R3 are likely to have a novel membrane trafficking system.

## Introduction

The sense of taste is both the guardian and guide for food intake, and it is vitally essential to animal body maintenance. In accordance with the environment, animal taste reception systems have undergone changes during the evolutionary process. For example, taste bud position varies among species: fish taste buds are located in the oral cavity and on the barbels, and insect taste buds are found on their lips and legs. Taste sensitivities also vary among species: carnivores have low sensitivity for sweet taste substances [1], [2], and some artificial sweeteners are detected by primates but not by rodents [3]–[5]. Since the identification of taste receptors, histological and structural biological studies have reported that the expression patterns of receptors and their binding systems vary among species [6]–[8].

In this study, we focused on the surface trafficking systems of human and mouse sweet taste receptors. These receptors exhibit unique properties and are capable of binding various substances, such as sugars, amino acids, peptides, and artificial sweeteners. Understanding these features requires structure-function analyses of sweet taste receptors, which are a known heterodimer of G protein-coupled receptors (GPCRs) T1r2/T1r3 [9]. GPCRs are the largest superfamily of membrane receptors and are involved in the regulation of numerous physiological functions. These integral membrane proteins share a common global topology comprised of seven transmembrane domains, an extracellular N-terminal domain, and an intracellular C-terminal domain and are classified in six large families (A–F) [10]. T1r2 and T1r3 are members of the T1r taste receptor family, which are considered class C GPCRs [9], [11], [12].

Class C GPCRs are characterized by a large extracellular amino-terminal domain called the Venus flytrap module (VFTM) containing a ligand-binding pocket. Representatives of class C GPCRs are metabotropic glutamate receptors (mGluRs), Ca^2+^-sensing receptors, V2r vomeronasal receptors, the gamma-aminobutyric acid B (GABA_B_) receptor, and T1r taste receptors. mGluRs and Ca^2+^-sensing receptors function as homodimers, whereas GABA_B_ receptor and T1r taste receptors function as heterodimers [13].

The GABA_B_ receptor is composed of two subunits, GABA_B1_ (GB1) and GABA_B2_ (GB2) [14]. Heterodimerization of GB1 and GB2 is required to generate functional receptors [14]. Cell surface trafficking of GB1 is regulated by an endoplasmic reticulum retention signal (RSR motif) located in the intracellular C-terminal region [15], whereas GB2 is located on the cell membrane surface [16]. It was suggested that this RSR motif of GB1 might be masked through a coiled-coil interaction with the intracellular tail of GB2, such that GB1 reaches the surface only when associated with GB2 [15]. Conversely, the heterodimerization process of T1r taste receptors is unclear. T1r taste receptors and GABA_B_ receptor share low homology (about 25%), and the T1r taste receptor has no obvious RSR motif in the C-terminal region. Moreover, from a structural biology viewpoint, there is limited information on the membrane trafficking system of T1r taste receptors, although the molecular taste reception system is beginning to be elucidated.

In the present study, we developed and studied tagged T1r2 and T1r3 and found differences between the cell trafficking of human T1R3 (hT1R3) and that of mouse T1r3 (mT1r3). In addition, we found that the VFTMs of human T1R2 (hT1R2) and hT1R3 must both be present for appropriate membrane trafficking of hT1R2/hT1R3 and that the cysteine-rich domain (CRD) of hT1R3 contains a region related to the inhibition of hT1R3 membrane trafficking. Our findings provide a novel perspective on evolutionary changes in taste reception system.

## Experimental Procedures

### Materials

#### Sweet substances

Sucralose was obtained from Sigma-Aldrich (St. Louis, MO), and cyclamate was obtained from Alfa Aesar (Lancashire, UK).

### Experimental Animals

Eight-week-old male C57BL/6NCrj mice (Charles River Laboratories Japan, Yokohama, Japan) were treated in accordance with the basic guidelines of the Ministry of Agriculture, Forestry, and Fisheries of Japan for laboratory animal study. The experiments using mice were approved by an institutional animal care and use committee (IACUC) of National Food Research Institute (NARO) (approval number: H18-063). The mice were bred in an IACUC-registered rearing facility. The mice were sacrificed with an overdose of pentobarbital sodium (Tokyo Chemical Industry Co., LTD, Tokyo, Japan), and their brain and tongue were extirpated and used for reverse transcription-polymerase chain reaction (RT-PCR). The extirpations were carried out in the laboratory registered with the IACUC of the NARO.

### Plasmid Construction

hT1R2 and hT1R3 complementary DNAs (cDNAs; GenBank accession nos. NM_152232 and NM_152228, respectively) were obtained from human genomic DNA (Promega, Madison, WI) with an overlapping PCR strategy [17]. Mouse T1r2 (mT1r2) and mT1r3 cDNAs (GenBank accession nos. NM_031873 and AB049994, respectively) were cloned with RT-PCR using mRNA from mouse (C57BL/6J) circumvallate papillae epithelium samples that were obtained as previously described [18]. Mouse mGluR1 cDNA was cloned with RT-PCR using mRNA from mouse brain. Epitope-tagged hT1R2, hT1R3, mT1r2, mT1r3, and their truncated or chimera mutants were constructed using an overlapping PCR strategy [17]. The signal peptides of mouse mGluR1 (1–32 amino acid residues: GenBank accession no. NM_016976, 402–497) were used for all mutants. The deduced signal peptides of hT1R2 (1–21 amino acid residues: GenBank accession no. NM_152232, 2–64), hT1R3 (1–22 amino acid residues: GenBank accession no. NM_152228, 1–66), mT1r2 (1–21 amino acid residues: GenBank accession no. NM_031873, 32–94), and mT1r3 (1–22 amino acid residues: GenBank accession no. AB049994, 33–98) were exchanged with that of mGluR1. c-Myc and FLAG tags were inserted after the mGluR1 signal peptide. The mutants for hT1R2 or mT1r2 were subcloned into a pEF-DEST51 vector (Life Technologies, Carlsbad, CA), and those for hT1R3 or mT1r3 were subcloned into a pEAK10 vector (Edge BioSystems Inc., Gaithersburg, MD). A previously described Gα16-gust44 construct [19] was obtained via overlapping PCR and subcloned into a pcDNA5/FRT vector (Life Technologies). Human Gα16 (GenBank accession no. NM_002068) was cloned using a cDNA library from human kidney (Life Technologies). Gαgust was obtained previously [20]. The sequences of the cloned receptors and mutants were verified through sequencing using a BECKMAN CEQ2000 DNA Analysis System (Beckman Coulter, Inc., Brea, CA).

### Cell Culture and Transfection

The Flip-In 293 cell line (Life Technologies) was cultured in Dulbecco’s modified Eagle’s medium (Sigma-Aldrich) supplemented with 10% fetal calf serum (Life Technologies), and incubated at 37°C in a 5% CO_2_ humidified incubator. Lipofectamine LTX (Life Technologies) was used to transfect plasmid DNA. For all experiments, 0.1 µg/cm^2^ plasmid DNA was used for transfection.

### Stable Cell Lines Expressing Mutated Receptors

Before the transfection with T1r2, T1r3, or both mutant vectors, Gα16-gust44 was transfected into Flip-In 293 cells to obtain a cell line that stably expressed the Gα16-gust44 protein (G16-gust44 cells). All stable cell lines for T1r2, T1r3, or both mutants were generated by transfecting constructed plasmids vectors into G16-gust44 cells. Cells were selected for antibiotic resistance to hygromycin (100 µg/mL; InvivoGen, San Diego, CA) for Gα16-gust44, blasticidin (10 µg/mL, InvivoGen) for T1r2, and puromycin (1 µg/mL, InvivoGen) for T1r3. Resistant colonies were combined, expanded, and used for all analyses.

### Immunocytochemistry

For live cell-surface labeling, stable cell lines or post-transfected cells (24 h) were incubated in phosphate-buffered saline (PBS) containing 15 mM NaN_3_ and polyclonal antibody to c-Myc tag (Cat No. C3956, Sigma-Aldrich) or polyclonal antibody to FLAG tag (Cat No. F7425, Sigma-Aldrich) at a 1∶200 dilution at room temperature for 1 h. The cells were then washed in PBS containing 15 mM NaN_3_ and incubated with PBS containing 15 mM NaN_3_ and Alexa Fluor 488-labeled anti-rabbit immunoglobulin G (IgG) (Cat. No. A-11070, Life Technologies) at a 1∶250 dilution at room temperature for 30 min. After washing with PBS containing 15 mM NaN_3_, the cells were observed under a fluorescence microscope (Leica Microsystems, Wetzlar, Germany). For permeabilized staining, stable cell lines or post-transfected cells (24 h) were fixed in 4% paraformaldehyde for 15 min at room temperature. The cells were blocked in 1% horse serum (Thermo Fisher Scientific Inc., Waltham, MA) diluted in PBS and incubated in 1% horse serum/PBS containing a 1∶500 dilution of polyclonal antibody to c-Myc tag or a 1∶500 dilution of polyclonal antibody to FLAG tag at room temperature for 1 h. The cells were then washed in PBS and incubated with PBS containing Alexa Fluor 488-labeled anti-rabbit IgG (1∶500) at room temperature for 30 min. After washing with PBS, the cells were observed under a fluorescence microscope.

### Flow Cytometry Analysis

Cell surface-expressed FLAG-tagged hT1R3 and mT1r3 and their mutants were detected with flow cytometry. Stable cell lines were washed twice with ice-cold FACS buffer (PBS containing 2% FCS (BioWest, Nuaillé, France), 0.1% NaN_3_), then incubated for 15 min on ice with a polyclonal antibody to FLAG tag (1∶200), washed thrice with FACS buffer, and incubated for 15 min with phycoerythrin-labeled anti-rabbit IgG (1∶250; Cat. No. 12-4739­81, eBioscience Co., San Diego, CA) on ice. Cells were simultaneously stained with 7-amino-actinomycin D to eliminate dead cells. Samples were quantified on a FACSCanto II (BD Biosciences, San Jose, CA), and data were analyzed with BD FACSDiva software (BD Biosciences). Relative surface expression of FLAG-tagged hT1R3, mT1r3 and their mutants were calculated as follows: MFI (mean fluorescence intensity) of each sample/MFI of negative control sample. MFI was analyzed in 10,000 cells. G16-gust44 cells that did not express T1rs were used as negative controls. The analyses were performed in triplicate, and the results were compared with one-way analysis of variance (ANOVA) followed by Tukey’s post hoc test of significance using the SigmaPlot software (Systat Inc., Chicago, IL).

### Western Blot Analysis

Stable cell lines were lysed with Cell Lysis Reagent, suitable for mammalian cell lysis and protein solubilization (Sigma-Aldrich) for 30 min on ice. After centrifugation (20,000×*g* for 10 min), the supernatant was mixed with lithium dodecyl sulfate sample loading buffer (Life Technologies). Part of the supernatant for the c-Myc-tagged protein was used for immunoprecipitation, which was carried out using a c-Myc-tagged Protein MILD PURIFICATION KIT Ver. 2 (Medical & Biological Laboratories Co. Ltd., Nagoya, Japan). Anti-c-Myc tag beads were added to the supernatant and incubated at 4°C for 1 h. After the beads were washed with PBS, the c-Myc-tagged protein was eluted with 1 mg/mL c-Myc tag peptide (Medical & Biological Laboratories Co. Ltd.) and used for western blot analyses. Sodium dodecyl sulfate-polyacrylamide gel electrophoresis (SDS-PAGE) and western blot analyses were performed using NuPAGE gel systems (Life Technologies). Membranes were incubated with monoclonal antibody to c-Myc tag (clone 9E10, Cat. No. M4439, Sigma-Aldrich) at a 1∶2000 dilution or a polyclonal antibody to FLAG tag at a 1∶2000 dilution and subsequently with horseradish peroxidase-linked anti mouse or rabbit IgG (Cat. No. NA931 or NA 934; GE Healthcare UK Ltd., Buckinghamshire, UK). ECL-Plus (GE Healthcare UK) was used to detect the target proteins on the membranes. The signals were observed with LightCapture (ATTO, Tokyo, Japan).

### Ca^2+^ Imaging

We performed 96-well plate Ca^2+^ flux assays using a FLEX station 3 (Molecular Devices, LLC, Sunnyvale, CA). Cells from the stable cell line (2.5×10^4^) were transferred onto a glass-based 96-well plate (Greiner Bio One, Frickenhausen, Germany). After 16 to 30 h, the wells were loaded with 100 µL Hank’s balanced salt solution (Sigma-Aldrich) containing 5 µM of the Ca^2+^ indicator dye Fluo8 NW (AAT Bioquest Inc., Sunnyvale, CA) and incubated for 60 min at 37°C. The stimulation was performed by adding 25 µL 5× concentrated solutions of sweet taste substances using a pipette. The intensity of the response was represented as the ratio (ΔF) relative to the baseline (F) and was plotted versus ligand concentration. The concentration–response curves were fitted to the Hill equation using the SigmaPlot software. The analyses were performed 3–6 times.

## Results

### Construction of Tagged T1r2/T1r3 Mutants to Evaluate Membrane-trafficked T1r2/T1r3

To reliably determine the T1r2/T1r3 surface trafficking system, we introduced the c-Myc or FLAG epitope in the N-terminal domains of hT1R2, hT1R3, mT1r2, and mT1r3 (CH2, FH3, Cm2, and Fm3, respectively). The signal peptide cleavage sites for T1r taste receptors have not been determined. Therefore, the signal peptide cleavage cites of T1r2 and T1r3 were speculated via homology searches among T1r2, T1r3, and mGluR1. Among the class C GPCRs, the mGluR1 signal peptide has been identified [21]. Prediction software is available for signal peptides; however, the determined signal peptide of mGluR1 was different from that predicted by the software. Therefore, we compared the N-terminal sequences among mGluR1, T1r2, and T1r3 receptors and deduced the 21- and 22-amino acid chains at the N-terminal regions of T1r2 (hT1R2 and mT1r2) and T1r3 (hT1R3 and mT1r3), respectively, as signal peptides.

**Figure 1:**
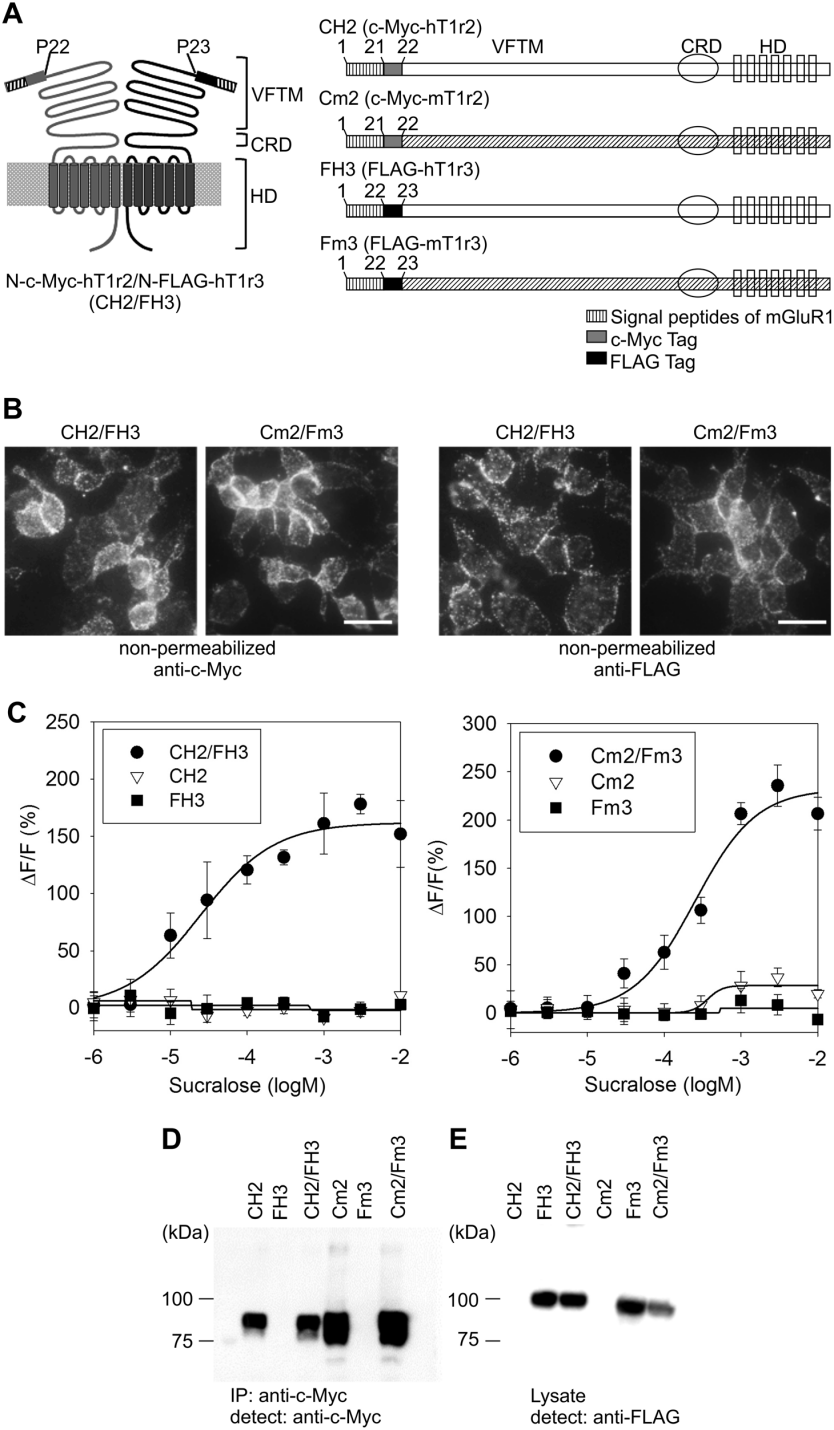
Construction of tagged mouse T1r2 and T1r3, and human T1R2 and T1R3. A. Schematic diagrams of tagged mouse T1r2 and T1r3, and human T1R2 and T1R3. The striped section corresponds to the signal peptide of mouse mGluR1, and the gray and black boxes represent c-Myc and FLAG tags, respectively. CH2, c-Myc-tagged human T1R2 (hT1R2); Cm2, c-Myc-tagged mouse T1r2 (mT1r2); CRD, cysteine-rich domain; FH3, FLAG-tagged human T1R3 (hT1R3); Fm3, FLAG-tagged mouse T1r3 (mT1r3); HD, heptahelical domain; VFTM, Venus flytrap modules. B. Surface expression of tagged mT1r2/mT1r3 and hT1R2/hT1R3. HEK293 cells stably expressing c-Myc-tagged T1r2 (hT1R2 and mT1r2) and FLAG-tagged T1r3 (hT1R3 and mT1r3) and labeled with rabbit anti-c-Myc antibody or rabbit anti-FLAG antibody under non-permeabilized conditions (scale bar = 50 µm). C. Tagged T1r2/T1r3s functions as a sweet taste receptor. The intensity of the response was represented as the ratio (ΔF) relative to the baseline (F) and was plotted versus ligand concentration. The cells expressing tagged T1r2/tagged T1r3 (filled circles) responded to sucralose. Error bars: SD (n = 3–6). D. Immunoblot analysis of cells expressing tagged T1r2s. The sample proteins were obtained through immunoprecipitation from 7.5×10^5^ cells/well using an anti-c-Myc antibody. E. Immunoblot analysis of cells expressing tagged T1r3 with a rabbit anti-FLAG antibody using cell lysate from 2.5×10^4^ cells/well.

We introduced a c-Myc or FLAG epitope downstream of the mGluR1 signal peptide cleavage site instead of that for T1r2 or T1r3 (Figure 1A). The tagged receptor genes were expressed in human embryonic kidney (HEK293) cells expressing a chimeric G protein, Gα16-gust44, which is reportedly a suitable Gα protein for T1r2 and T1r3 in sweet taste response analyses using Ca^2+^ imaging [7]. The surface trafficking capabilities of T1r2/T1r3 were determined with immunolabeling under non-permeabilized conditions. The cells stably coexpressing tagged hT1R2/hT1R3 (CH2/FH3) or tagged mT1r2/mT1r3 (Cm2/Fm3) were labeled with an anti-c-Myc or anti-FLAG antibody under non-permeabilized conditions (Figure 1B), suggesting that both CH2/FH3 and Cm2/Fm3 were properly expressed on the membrane surface. Responses to sucralose were observed in both CH2/FH3 and Cm2/Fm3 cells (Figure 1C), whereas the sensitivity of CH2/FH3 for sucralose was higher than that of Cm2/Fm3: the half-maximal effective concentrations were 2.7×10^−5^ M and 3.00×10^−4^ M, respectively.

Using signal peptides from hT1R2 and hT1R3 yielded similar membrane trafficking and responses to sweet taste substances, whereas the expression level of these mutants was lower than those obtained with mGluR1 signal peptides (Figure S1). For these experiments, we performed western blots to confirm the expression levels of the T1r2 and T1r3 mutants (Figure 1D, E). FLAG-tagged hT1R3 and mT1r3 were easily detected when whole-cell lysates were used, whereas c-Myc-tagged hT1R2 and mT1r2 were only detected following immunoprecipitation. This is not due to the tag because c-Myc-tagged hT1R3 was detected when the whole-cell lysate was used (Figure S2).

### Differences in Cell Trafficking Capabilities between hT1r3 and mT1r3

**Figure 2:**
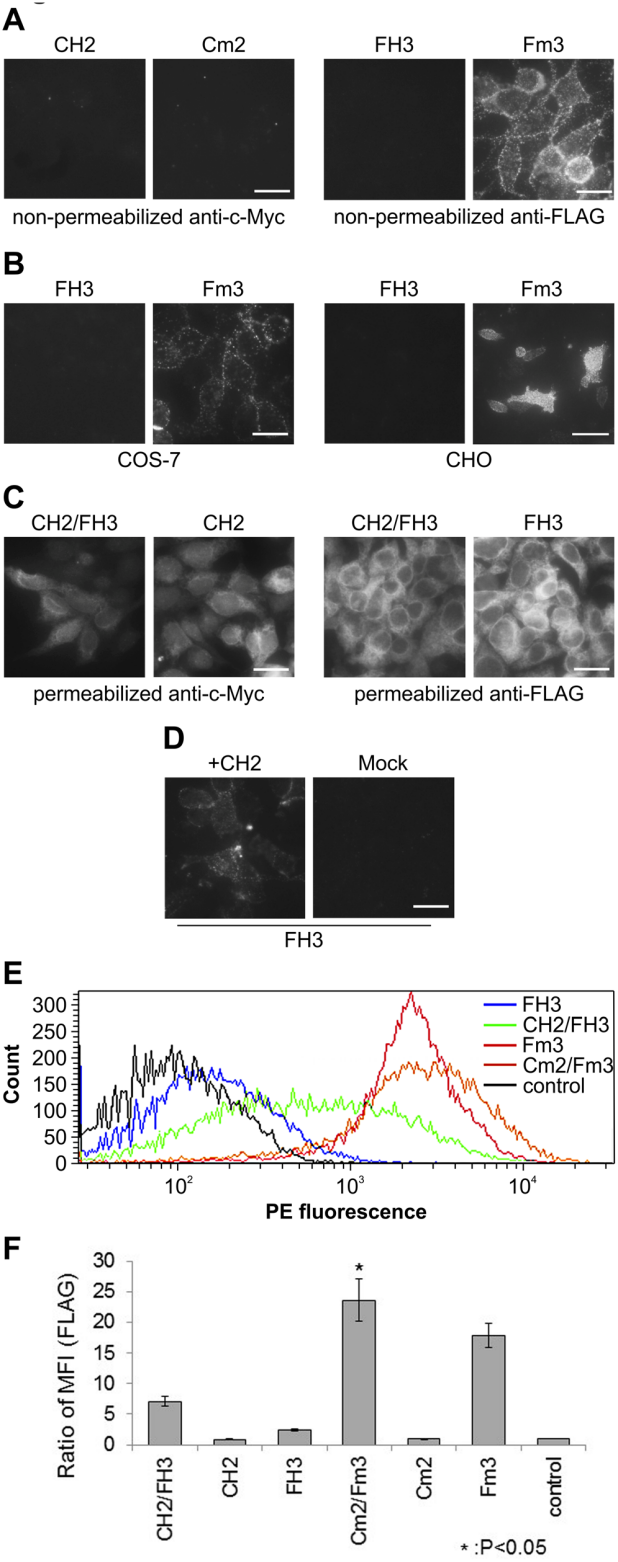
Surface expression of tagged T1r2 and T1r3. A. Surface expression of tagged T1r2 and T1r3 solely in HEK293 cells. HEK293 cells stably expressing tagged T1r2 or tagged T1r3 were labeled with a rabbit anti-c-Myc antibody or rabbit anti-FLAG antibody under non-permeabilized conditions (scale bar = 50 µm). B. Surface expression of tagged T1r3 in COS-7 and CHO cells. Tagged T1r3 were transiently expressed in COS-7 or CHO cells and were labeled with a rabbit anti-FLAG antibody under non-permeabilized conditions (scale bars = 50 µm) C. Tagged T1rs expression in HEK293 cells. The cells were labeled with a rabbit anti-c-Myc antibody or rabbit anti-FLAG antibody under permeabilized conditions (scale bar = 50 µm). D. Surface expression of FH3 with transient CH2 expression. FH3 cells were labeled with a rabbit anti-FLAG antibody under non-permeabilized conditions after 24-h lipofection with CH2 (scale bar = 50 µm). E. Representative flow cytometry histogram data. HEK293 cells were used as a control. F. Flow cytometry quantification of cell surface expression of tagged T1r3 in intact cells. Data are expressed as the mean fluorescence intensity (MFI) ratio of FLAG labeling (MFI [FLAG]) in cells expressing tagged mouse T1r2, T1r3, T1r2/T1r3, human T1R2, T1R3, or T1R2/T1R3 to that in control HEK293 cells. Statistical significance was calculated by ANOVA followed by Tukey test (*: P<0.05). Error bars: SEM (n = 3).

Next, the surface trafficking capabilities of tagged T1r2 and T1r3 (CH2, FH3, Cm2, Fm3) were analyzed with immunolabeling and flow cytometry. Cells stably expressing CH2, FH3, Cm2, or Fm3 were stained with an anti-c-Myc or anti-FLAG antibody under non-permeabilized conditions. Interestingly, tagged hT1R3 (FH3) did not result in surface labeling above background levels, whereas tagged mT1r3 (Fm3) was labeled by the FLAG-tag antibody (Figure 2A). Surface labeling levels for tagged hT1R2 (CH2) and tagged mT1r2 were not observed (see Figure 2A). Flow cytometry analysis using an anti-FLAG antibody revealed that CH2/FH3 cells had greater cell surface FLAG tag expression compared with that of FH3 cells (Figure 2E, F). We produced each stable cell line without selecting particular colonies to avoid biased results. Thus, the flow cytometry histogram revealed a wide variety of CH2/FH3 labeling intensities, probably because the diverse hT1R2 expression levels affected hT1R3 surface expression levels in CH2/FH3 cells (Figure 2E). The labeling intensities against FLAG tag in Cm2/Fm3 and Fm3 cells showed an MFI that was two- to threefold higher than that in CH2/FH3 cells (Figure 2F). Poor surface expression levels of CH2, FH3, and Cm2 were not explained by the total receptor expression levels in HEK293 cells because the expression of these tagged receptors was detected with c-Myc or FLAG antibody under permeabilized conditions (Figure 2C), and no apparent difference was observed by western blot analysis (Figure 1D, E). The same results were obtained when FH3 and Fm3 were expressed in monkey COS-7 cells and Chinese hamster ovary (CHO) cells (Figure 2B). These findings suggested that membrane trafficking was not dependent on cell origin. To confirm hT1R3 expression and function, we labeled FH3 cells transiently expressing CH2 with anti-FLAG antibody under non-permeabilized conditions. As expected, FH3 expression was only detected on the membrane when CH2 was expressed (Figure 2D). This result suggested that the same results were obtained with both stable and transient expression systems.

### Identification of the Membrane Trafficking Region of hT1r3

**Figure 3:**
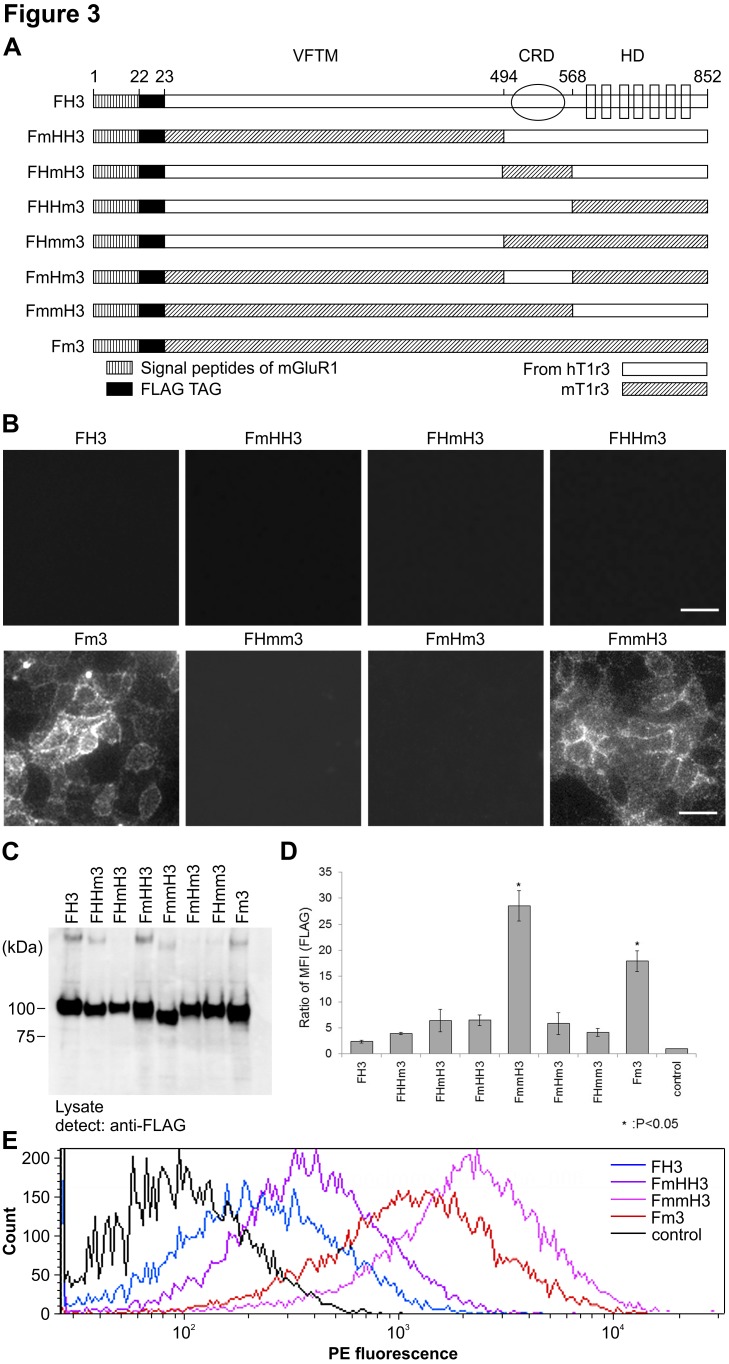
Surface expression of human-mouse T1r3 chimeras. A. Construction of T1r3 chimeras. The mouse regions are shaded in grey. B. Surface expression of T1r3 chimeras. HEK293 cells expressing chimeras were labeled with rabbit anti-FLAG antibody under non-permeabilized conditions (scale bar = 50 µm). C. Immunoblot analysis of cells expressing chimeras with rabbit anti-FLAG (2.5×10^4^ cells/well). D. Flow cytometry quantification of cell surface expression of chimeras in intact cells. Data are expressed as the MFI ratio of FLAG labeling (MFI [FLAG]) in chimera-expressing cells. Statistical significance was calculated by ANOVA followed by Tukey test (*: P<0.05). Error bars: SEM (n = 3). E. Representative flow cytometry histogram data.

**Figure 4:**
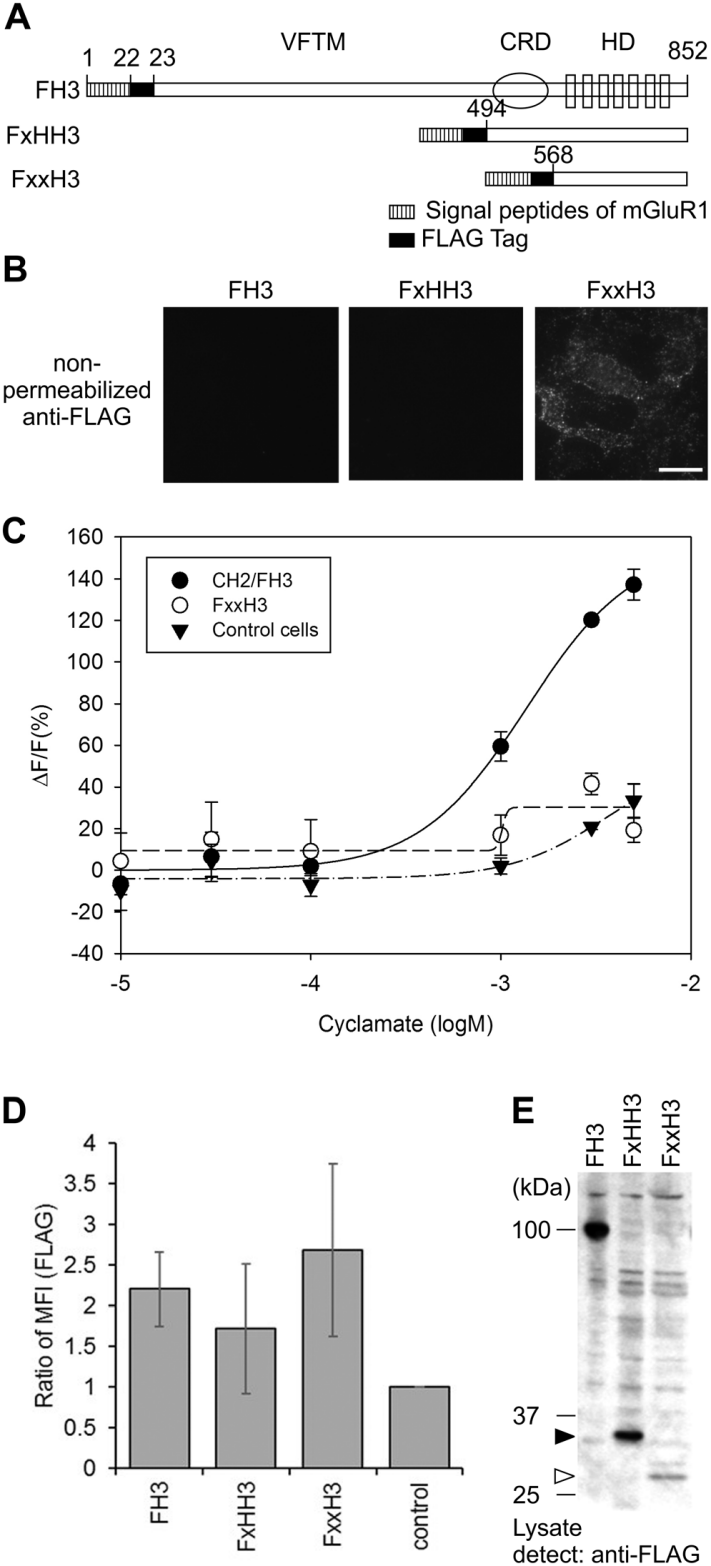
Surface expression of truncated human T1R3 mutants. A. Construction of truncated human T1R3 mutants. All mutants were tagged with a FLAG epitope under the mGluR1 signal peptide. B. Truncated mutant surface expression. HEK293 cells expressing mutants were labeled with a rabbit anti-FLAG antibody under non-permeabilized conditions (scale bar = 50 µm). C. The responses of truncated mutants for the sweet taste substance cyclamate. FxxH3-expressing cells (open circles) or control cells (filled triangles) showed little response to cyclamate, whereas cells expressing hT1R2/hT1R3 (filled circles) did respond to cyclamate. Error bars: SD (n = 3–6). The intensity of the response was represented as the ratio (ΔF) relative to the baseline (F) and was plotted versus ligand concentration. D. Flow cytometry quantification of cell surface expression of truncated mutants in intact cells. Data are expressed as the MFI ratio of FLAG labeling (MFI [FLAG]) in chimera-expressing cells. Error bars: SEM (n = 3). E. Immunoblot analysis of truncated mutants treated with a rabbit anti-FLAG (7.5×10^4^ cells/well). The filled arrowhead indicates a molecular weight of approximately 33 kDa for FxHH3, and the open arrowhead indicates approximately 27 kDa for FxxH3.

To identify the regions in hT1R3 that regulate its trafficking to the plasma membrane, we analyzed the membrane trafficking capabilities of domain-swapped chimera mutants of hT1R3 and mT1r3 (Figure 3A). These chimera mutants were obtained by swapping the VFTM, CRD, and heptahelical domain (HD) from hT1R3 and mT1r3. FLAG tags were added to each chimera and stably expressed in HEK293 cells (Figure 3C) to evaluate cell surface trafficking. When the cell lines were labeled with anti-FLAG antibodies under non-permeabilized conditions (Figure 3B), the chimera FmmH3, in which the VFTM and CRD were from mT1r3 and the HD was from hT1R3, was labeled, but the other chimeras were weakly or not labeled. Flow cytometry analysis using an anti-FLAG antibody also showed that FmmH3 and Fm3 cells exhibited significantly higher cell-surface FLAG tag expression (Figure 3D, E). We then analyzed the membrane trafficking capabilities of truncated mutants of hT1R3 (Figure 4) obtained by removing the VFTM, CRD, or both from FLAG-hT1R3 (Figure 4A) and adding FLAG tags. Interestingly, surface expression was faintly observed on fluorescence microscopy when only the N-terminal-FLAG-tagged HD of hT1R3 was stably expressed in HEK293 cells (FxxH3 cells, Figure 4B). Conversely, flow cytometry analysis did not show significant differences among the MFIs for FH3, FxHH3, and FxxH3 (Figure 4D), and the obvious response for cyclamate was not observed (Figure 4C). Western blot analysis revealed that the expression level for FxxH3 was apparently low compared with that for FH3 and FxHH3 (Figure 4E). The results obtained from chimera and truncated mutants raise the possibility that the VFTM and CRD of hT1R3 contained the region that inhibited hT1R3 surface expression.

### VFTMs of hT1r2 and hT1r3 are Required for Membrane Trafficking and Sweet Taste Response

**Figure 5:**
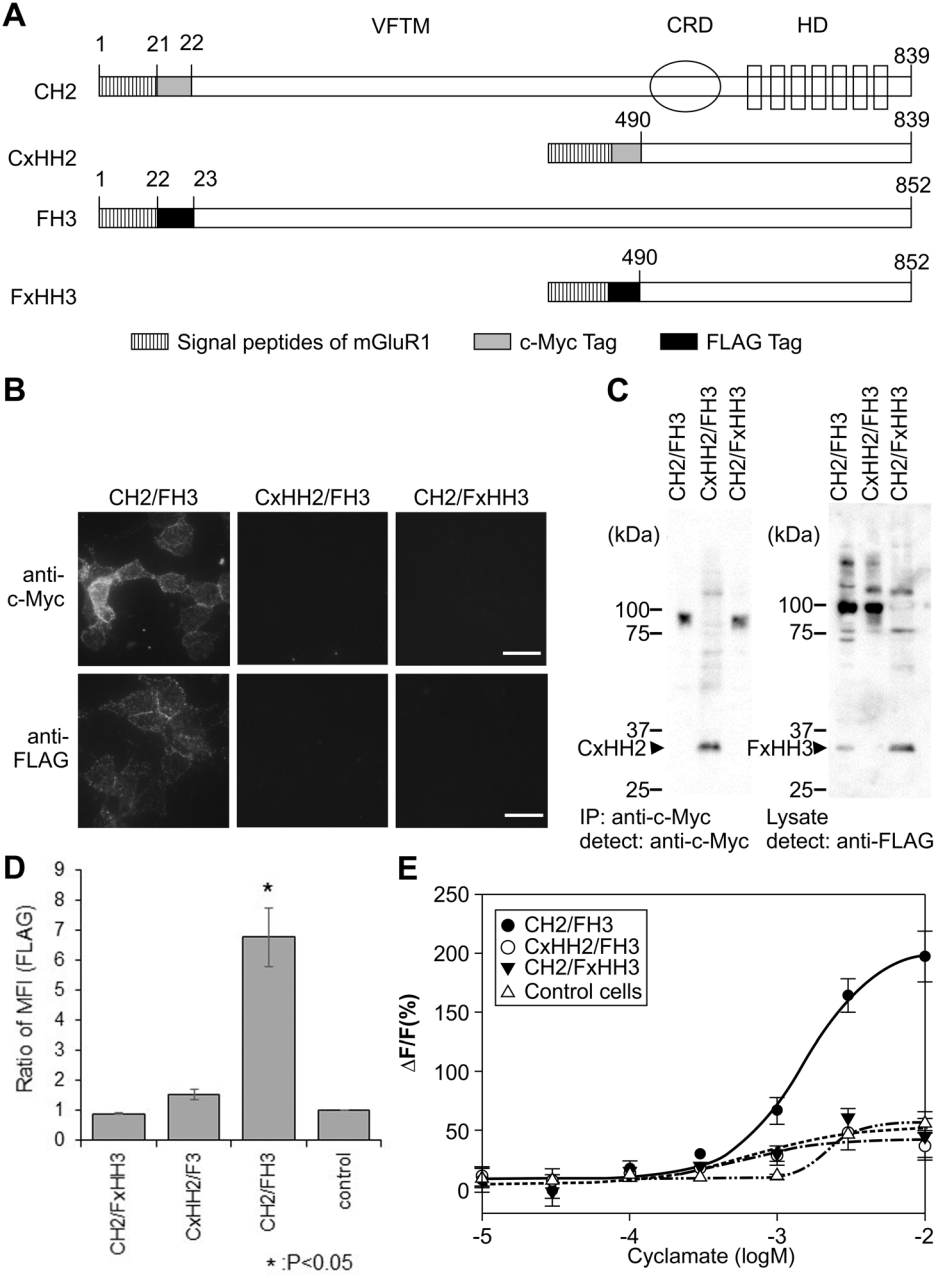
Surface expression of VFTM-truncated human T1R2/T1R3 mutants. A. Construction of the truncated mutants of human T1R2 and T1R3. All mutants were tagged with c-Myc or FLAG epitopes under the mGluR1 signal peptide. B. Surface expression of the truncated mutants. HEK293 cells expressing mutants were labeled with a rabbit anti-c-Myc antibody or rabbit anti-FLAG antibody under non-permeabilized conditions (scale bar = 50 µm). C. Immunoblot analysis of truncated mutant-expressing cells. (Left panel) The sample proteins for truncated hT1R2 (CxHH2) or c-Myc tagged hT1R2 (CH2) were obtained through immunoprecipitation from 7.5×10^5^ cells/well using an anti-c-Myc antibody. (Right panel) The cell lysates from 2.5×10^4^ cells/well were used for truncated hT1R3 (FxHH3) or FLAG tagged hT1R3 (FH3) proteins. Arrowheads indicate molecular weights of approximately 33 kDa for both CxHH2 and FxHH3. D. Flow cytometry quantification of cell surface expression of the truncated mutants. Data are expressed as the MFI ratio of FLAG (MFI [FLAG]) labeling in mutant-expressing cells. Statistical significance was calculated by ANOVA followed by Tukey test (*: P<0.05). Error bars: SEM (n = 3). E. Responses of truncated T1rs to cyclamate. Cells expressing CxHH2/FH3 (open triangles) or CH2/FxHH3 (filled squares) showed little response to cyclamate, whereas cells expressing CH2/FH3 (filled circles) did respond to cyclamate. The intensity of the response was represented as the ratio (ΔF) relative to the baseline (F) and was plotted versus ligand concentration. Error bars: SD (n = 3–6).

Our analysis showed that FH3 alone could not traffic to the cell membrane, whereas CH2/FH3 could. These data suggest that interaction with hT1R2 is required for hT1R3 membrane trafficking. Based on the chimeric mutant data (Figure 3), we hypothesized that the important region for membrane trafficking of hT1R3 was in the VFTM, CRD, or both. Therefore, we analyzed the membrane trafficking capabilities of truncated mutants of hT1R2 with hT1R3 and mutants of hT1R3 with hT1R2. These mutants were obtained by removing the VFTM from c-Myc-tagged hT1R2 and FLAG-tagged hT1R3 (CxHH2 and FxHH3, Figure 5A) and expressing them in FH3 or CH2 cell lines (Figure 5C). These cell lines (CxHH2/FH3, CH2/FxHH3) were labeled with anti-c-Myc or anti-FLAG antibodies under non-permeabilized conditions. Coexpression of these hT1R2 or hT1R3 mutants with hT1R3 or hT1R2 revealed that hT1R2/hT1R3 surface expression was diminished by the truncated VFTM of hT1R2 or hT1R3 (Figure 5B, D).

Cyclamate is a sweetener that binds the HD of hT1R3 [7], whereas sucralose has been deduced to bind the VFTM of hT1R2 [22]. We used cyclamate for analyses of response to sweet taste substances in the CxHH2/FH3 and CH2/FxHH3 cell lines because the deletion of the VFTM in hT1R2 led to the loss of the sucralose binding site. The loss of membrane trafficking diminished the sweet taste responses of the truncated VFTMs of hT1R2 or hT1R3, and their responses to cyclamate were not observed (Figure 5E).

## Discussion

In this study, we identified a unique membrane trafficking system for hT1R3 and determined that the coexpression of hT1R2 and hT1R3 is required for its membrane trafficking in heterologous expression systems.

### Validity of the Evaluation System for of T1r2/T1r3 Membrane Trafficking

We first constructed the tagged T1r2 or T1r3 mutants in this study to evaluate T1rs membrane trafficking. We were able to deduce the previously unknown signal peptides of T1rs and alter them to that of mGluR1. Our T1r2/T1r3 mutant responded to sweet taste substances, suggesting that the deduced signal peptides might be appropriate. However, the true signal peptides for T1r2 and T1r3 are still unknown and remain a focus of study. In our study, it was difficult to detect T1r2 expression by western blotting; it could only be detected through immunoprecipitation. This outcome was unchanged with T1r3 coexpression. On the other hand, T1r2 expression in and on the HEK293 cells was detected by immunocytochemistry and was not obviously different from that of T1r3. Based on these results, we speculate that the T1r2 protein was unstable in the lysate solution, which contained a detergent. Improving T1r2’s stability in the lysate buffer will be important for future studies of T1r2 function.

### Difference in hT1r3 and mT1r3 Trafficking

Our results clearly demonstrated a difference in membrane trafficking between hT1R3 and mT1r3. There are only a few reports on molecules that display membrane trafficking variation depending on species; for example, the membrane trafficking systems of serotonin-2a receptor [23] and gonadotropin-releasing hormone receptor [24] vary between rodents and human. We used the mGluR1 signal peptide for all chimeric constructions, suggesting that the hT1R3 and mT1r3 signal peptides do not account for their variable membrane trafficking capabilities. While the biological significance of differential membrane trafficking between hT1R3 and mT1r3 was unclear, it is known that the response of hT1R2/hT1R3 to sweet taste substances is different from that of mT1r2/mT1r3 [25]. Some artificial sweet substances, such as aspartame and cyclamate, can bind to hT1R2/hT1R3 but not to mT1r2/mT1r3 [7], [26]. Mammals are known to have evolved with a variety of taste sensitivities depending on their feeding environment, leading to speculation that taste receptors might exhibit variations in both the interaction sites of sweet taste substances and in membrane trafficking capabilities. Further examination of the relationship between functional evolution and dietary environment is needed to answer these questions.

### Coordination between CRD and VFTM for hT1R3 Membrane Trafficking

Our results showed that mutants FmmH3 and FxxH3 trafficked to the cell membrane, whereas FmHH3 and FxHH3 did not. These results suggested that the hT1R3 CRD prevents its membrane trafficking. This study is the first to demonstrate that CRDs in class C GPCRs are involved in membrane trafficking. We found that FmHm3 membrane trafficking was extremely weak compared with that of Fm3, suggesting that the CRD is essential for hT1R3 membrane trafficking. However, Hu et al. [27] demonstrated that deletion of the entire CRD did not significantly affect the surface expression of Ca^2+^-sensing receptor, and FHmH3, which contains a CRD from mT1r3, did not traffic to the cell membrane, suggesting that the CRD alone might not be a general critical region for membrane trafficking of class C GPCRs. The CRD plays a role in signal transmission for the Ca^2+^ ion receptor [27], and the CRD in mGluR1 is implicated in its expression and ability to bind ligands [28]. Moreover, GABA_B_ receptors do not possess a CRD. It has been suggested that the CRD of hT1R2/hT1R3 contains a binding site for the sweet protein brazzein [29]; however, there is insufficient information regarding the function of the CRD in class C GPCRs, and further studies are needed. We found that FmmH3 trafficked to the cell membrane, whereas FmHm3 and FHmm3 did not. These results indicated that both the VFTM and the CRD of hT1R3 might function coordinately as inhibitory sites for hT1R3 surface expression, but it is possible that there is only one such site in hT1R3. We also considered that the site was located on the boundary between the VFTM and the CRD; however, determining the site is difficult because neither the VFTM nor the CRD contains a consensus motif. Therefore, we did not determine the number of critical sites for membrane traffic in hT1R3, and it remains largely speculative.

### The VFTMs of hT1R2 for hT1r2/hT1r3 Membrane Trafficking

Our results suggested that VFTMs are necessary for hT1R2/hT1R3 membrane trafficking. This requirement may indicate a novel membrane trafficking system among class C GPCRs. Compared with those of the GABA_B_ receptor, another heterodimeric class C GPCR, the properties of the membrane trafficking system of hT1R2/hT1R3 are quite different.

In GABA_B_ receptors, the retention signal is located in the C-terminal region of the GB1 subunit and is probably shielded by the C-terminal coiled-coil interaction of the GB1 and GB2 subunits [30]. The C-terminal region of the GABA_B_ receptor that includes this retention signal is unrelated to heterodimerization [15], [30]. Conversely, at least three regions are related to the membrane trafficking of hT1R2 and hT1R3: the VFTM of hTr2, the VFTM of hT1R3, and the CRD of hTr3. Many sweet taste substances reportedly bind to the VFTM of hT1R2 [22], and a sweet taste-modified substance binds the VFTM of hT1R3 [31], suggesting that the VFTMs of hT1R2 and hT1R3 are responsible for both the reception of sweet taste substances and receptor membrane trafficking. The C-terminal region of hT1R3 might not contribute to the difference in cell membrane trafficking between mT1r3 and hT1R3, because the surface expression of Fm3 was nearly equal to that of FmmH3, in which the HD-containing C-terminal of the region was exchanged with human version. This result does not disprove that the C-terminal region contains cell membrane traffic system-related sites. If the C-terminal region of T1r3 does contain such sites, it may be a common membrane trafficking system across species, and this should be investigated in the future.

### Relationship between Membrane Trafficking and the Sweet Taste Response of T1r2/T1r3

In this study, we also analyzed the relationship between receptor surface expression and the response to sweet substances when T1r2/T1r3 and T1r3 were mutated. No obvious response was detected when hT1R2, hT1R3, mT1r2, or mT1r3 were expressed alone in HEK293 cells. The sweet response of mT1r3 located at the cell surface was quite small, as was that of intracellular hT1R3, suggesting that homodimerization of mT1r3, even if it exists, might not result in a properly functioning sweet taste receptor.

mGluRs and hT1R2/hT1R3 displayed different properties. mGluRs can only function when their HDs are expressed [32]. The HDs of mGluRs are highly expressed on the membrane surface and form and function properly without the VFTM and CRD. Conversely, although the HD of hT1R3 is also sufficient to facilitate membrane traffic, its expression level is very low, and it did not show distinct response to cyclamate, which is the known ligand to the transmembrane domain of hT1R3 (FxxH3). These data indicated that the HD of hT1R3 could not form and function properly, however, there remains a possibility that low FxxH3 expression may prevent the measurement of responses to cyclamate.

In conclusion, our results demonstrate that the structure and function of T1r2/T1r3 vary among species. Both the interaction sites of sweet taste substances and membrane trafficking capabilities were different for mice and human receptors. Investigation of the membrane trafficking systems of other species will improve our understanding of taste reception system evolution and the relationship between environment and food preferences.

## Supporting Information

Figure S1 **Tagged hT1R2 and hT1R3 using signal peptides from hT1R2 and hT1R3.** A. Surface expression of the tagged hT1R2 and hT1R3 using hT1R2 and hT1R3 signal peptides. Mutant-expressing HEK293 cells were labeled with a rabbit anti-FLAG antibody under non-permeabilized conditions (scale bar = 50 µm). B. Immunoblot analysis of cells expressing hT1R3 using signal peptides from hT1R3 with or a rabbit anti-FLAG antibody (2.5×10^4^ cells/well).
(TIF)

Figure S2 **The expression of c-Myc-tagged hT1R3.** Immunoblot analysis of cells expressing c-Myc-tagged hT1R2 and hT1R3 with a mouse anti-c-Myc antibody. Lane 1: The lysate of HEK293 cells expressing c-Myc-tagged hT1R2 (1×10^5^ cells/well). 2: The immunoprecipitation sample for c-Myc-tagged hT1R2 protein (1×10^6^ cells/well). 3: The lysate of samples for HEK293 cells expressing c-Myc-tagged hT1R3 (1×10^4^ cells/well).
(TIF)
