# Supplementary figures and images for: Distinct Human and Mouse Membrane Trafficking Systems for Sweet Taste Receptors T1r2 and T1r3
Source: PLoS One. 2014 Jul 16;9(7):e100425. doi: 10.1371/journal.pone.0100425 (PMC4100762; doi:10.1371/journal.pone.0100425)

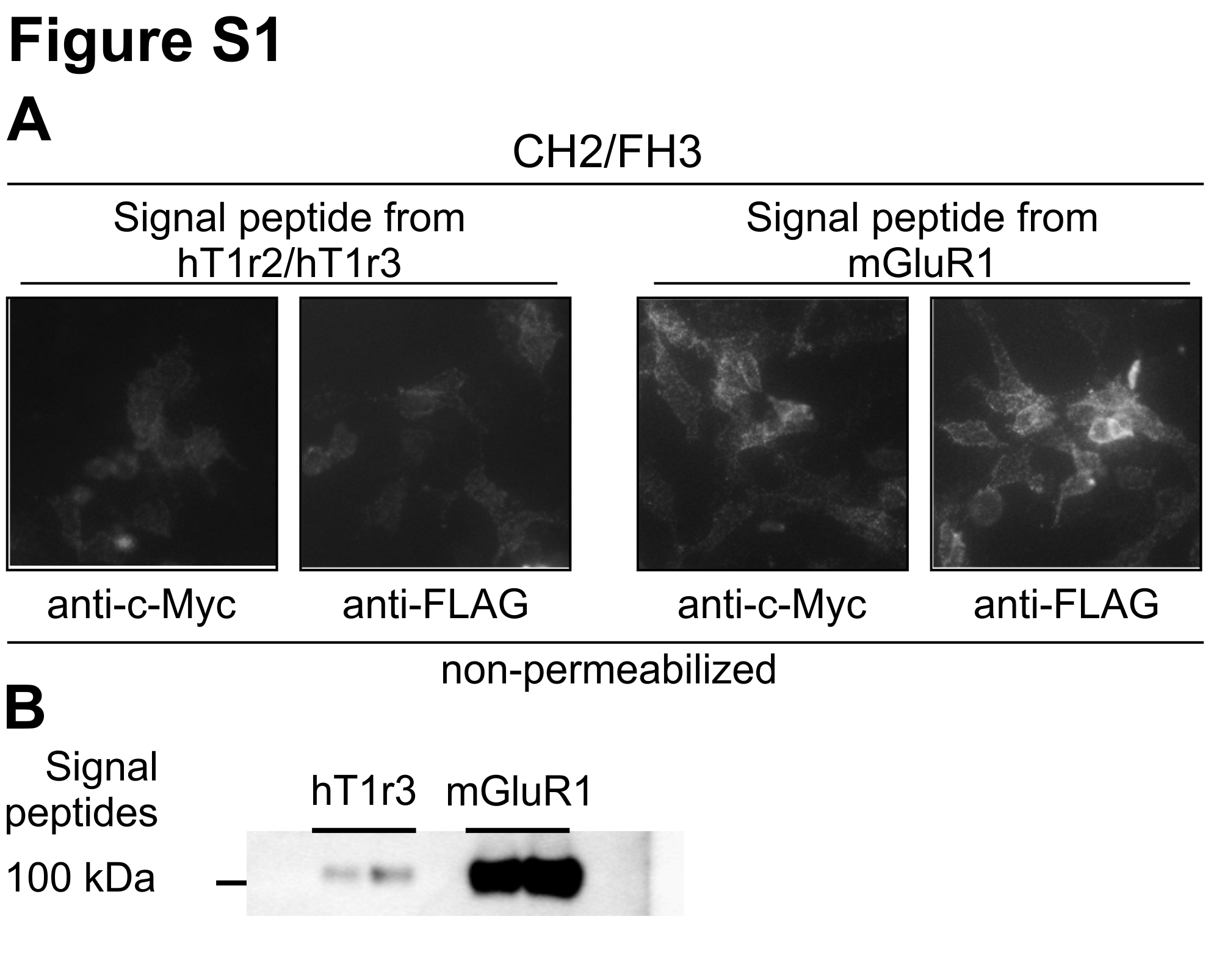

Supplement: Figure S1 — Tagged hT1R2 and hT1R3 using signal peptides from hT1R2 and hT1R3. A. Surface expression of the tagged hT1R2 and hT1R3 using hT1R2 and hT1R3 signal peptides. Mutant-expressing HEK293 cells were labeled with a rabbit anti-FLAG antibody under non-permeabilized conditions (scale bar = 50 µm). B. Immunoblot analysis of cells expressing hT1R3 using signal peptides from hT1R3 with or a rabbit anti-FLAG antibody (2.5×10^4^ cells/well). (TIF) [file pone.0100425.s001.tif]

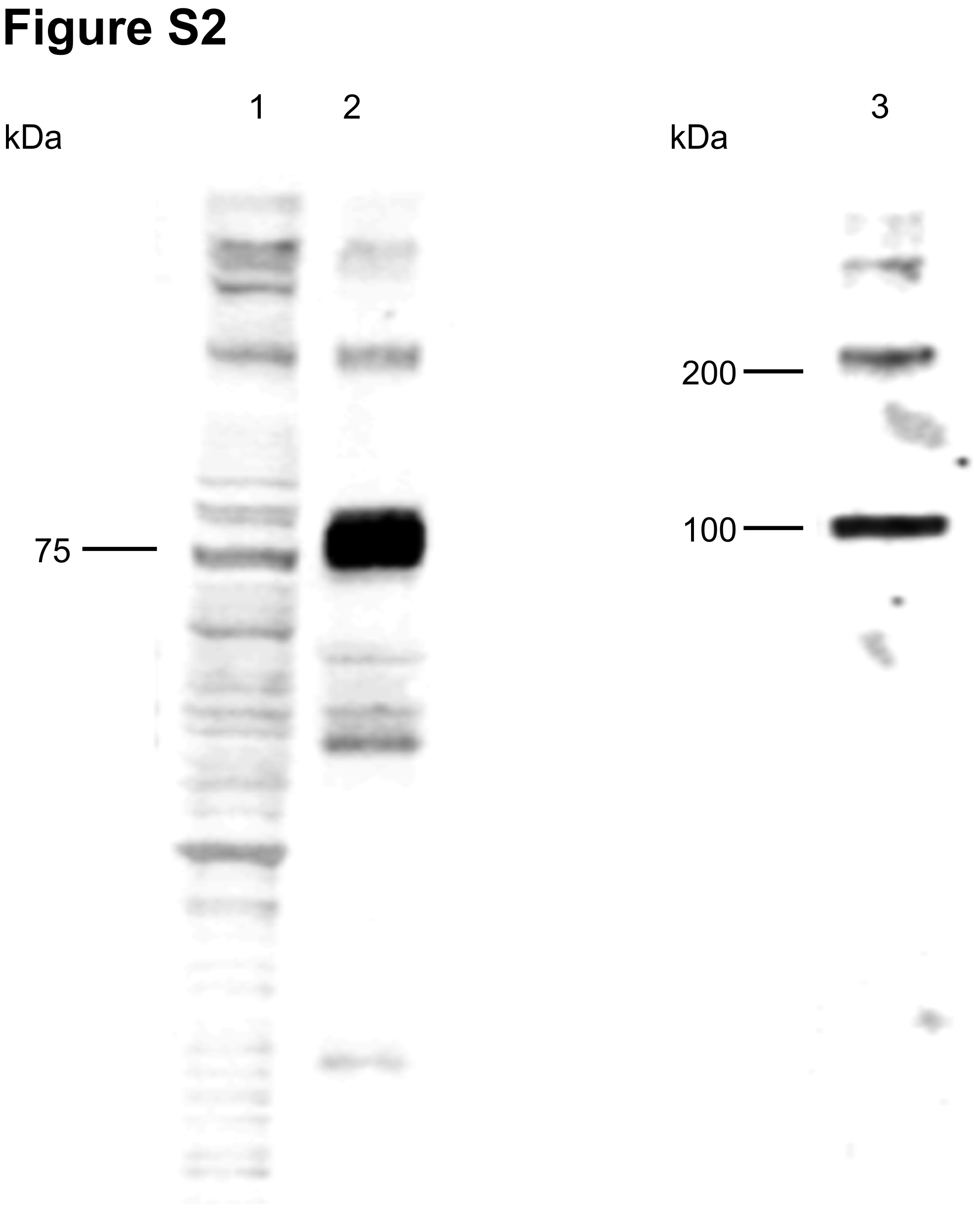

Supplement: Figure S2 — The expression of c-Myc-tagged hT1R3. Immunoblot analysis of cells expressing c-Myc-tagged hT1R2 and hT1R3 with a mouse anti-c-Myc antibody. Lane 1: The lysate of HEK293 cells expressing c-Myc-tagged hT1R2 (1×10^5^ cells/well). 2: The immunoprecipitation sample for c-Myc-tagged hT1R2 protein (1×10^6^ cells/well). 3: The lysate of samples for HEK293 cells expressing c-Myc-tagged hT1R3 (1×10^4^ cells/well). (TIF) [file pone.0100425.s002.tif]
